# Musculoskeletal pain in health professionals at the end of their studies and 1 year after entry into the profession: a multi-center longitudinal questionnaire study from Switzerland

**DOI:** 10.1186/s12891-023-06635-z

**Published:** 2023-06-23

**Authors:** Thomas Bucher, Thomas Volken, Fabian Pfeiffer, René Schaffert

**Affiliations:** Zurich University of Applied Sciences ZHAW, School of Health Sciences, Katharina-Sulzer-Platz 9, CH-8400 Winterthur, Switzerland

**Keywords:** Musculoskeletal pain, Back, Neck, Hand, Foot, Health professionals, Longitudinal

## Abstract

**Background:**

Musculoskeletal pain, especially back pain, is common among health care professionals (HP). For prevention purposes, it is important to know whether HP develop their symptoms before or after entering the health care workforce. Cross-sectional studies among HP cannot answer this question. This follow-up study measures the prevalence and individual course of musculoskeletal pain among full-time HP students at the end of their studies and one year after entering the health care workforce.

**Method:**

Self-reported one-year prevalence for low back pain, neck/shoulder pain, pain in arms/hands, and pain in legs/feet was collected at two timepoints from 1046 participating HP using an online questionnaire. Participants were asked whether their musculoskeletal pain was related to study or work conditions. Generalized estimating equation (GEE) models of the binomial family with log link were used to estimate adjusted prevalence and corresponding normal based 95% confidence intervals were derived using the bootstrap method with 1000 replications.

**Results:**

The prevalence of low back pain as well as neck and shoulder pain was very high at baseline and follow-up in all full-time students and later HP. Prevalence for pain in arms/hands, legs/feet was low and there were significant differences between the professions. HP clearly associated their low back pain and neck/shoulder pain with study and work conditions; HP strongly associated pain in arms/hands, legs/feet only with work conditions.

**Conclusion:**

Many HP suffer from back/neck/shoulder pain already as students before starting their professional career. The prevention of back/neck/shoulder pain must be part of the education of all health professions at universities. As an example of best practice, universities should incorporate ergonomic measures and exercises into the daily routine of training health professionals. The effects of physically demanding professional tasks on the upper and lower extremities need to be investigated in further studies to take preventive measures.

## Background

Musculoskeletal health is a key factor for human functioning, enabling mobility, dexterity, and the ability to work [1]. Low back pain, neck pain, and other musculoskeletal disorders are the leading cause of years lived with disability, with low back pain having the greatest impact worldwide [2]. Compared to other noncommunicable diseases, musculoskeletal disorders are the leading cause of years of productive life lost in the workforce [1]. In order to initiate and promote preventive and mitigating public health measures, it is important to identify populations at risk and to understand the causes and the development of musculoskeletal disorders in these populations.

Recent studies have reported a high prevalence of musculoskeletal disorders in students [3–6] and in health professionals (HP) [7–12]. In the absence of long-term studies in this population, it is uncertain whether HP develop their musculoskeletal disorders during their working lives or whether they were preexisting.

Therefore, we present this observational, follow-up study to investigate the prevalence and individual course of low back pain, neck/shoulder pain, pain in arms/hands, and pain in legs/feet in full-time HP at the end of their university studies (HP students) and 1 year later, after working as HP in the health care system. There are good reasons to investigate musculoskeletal disorders in young health professionals at the transition from study to work.

Given the shortage of qualified HP, it is important to integrate and retain young HP in the health care workforce by taking care of their health as early as possible. Studies show that work-related and chronic musculoskeletal disorders may be a reason for HP students to discontinue their studies [13, 14], reduce the ability to perform job tasks and roles [15, 16], and lead to reduced productivity when people attend work despite disorders (presenteeism) [17]. Musculoskeletal disorders also predict burnout [18], lead to sickness absence and often to long-term absence (absenteeism) [19], and cause HP to change their specialty or role at work or to leave the profession [3, 15, 20].

Young HP in the transition from study to work are predominantly female and between 20 and 30 years old. Low back pain is most prevalent in this age group [3] and women are more prone to neck pain than men [3]. The first onset of work-related upper limb symptoms is also common among HP within the first 5 years of work [3].

### Research questions

Most studies of HP and HP students measure musculoskeletal disorders at only one point in time. As a result, there is no evidence on whether musculoskeletal disorders are acquired in the health care workplace or occur before. This information is crucial for the prevention of musculoskeletal disorders in future HP. Therefore, in this longitudinal study we investigate the following questions:

1. The prevalence and individual dynamics of low back pain, neck pain, pain in arms/hands, and pain in legs/feet among full-time HP students at the end of their studies (baseline) and 1 year later after entering the health care workplace (follow-up).
2. Differences in the prevalence of musculoskeletal pain among students/professionals of occupational therapy, nutritional sciences, midwifery, nursing and physiotherapy.
3. The causal attributions HP make for their pain.

## Methods

### Study design

This study is a multi-center, follow-up study with two measurement points. Baseline data were collected from full-time HP students (occupational therapy, nutritional sciences, midwifery, nursing, and physiotherapy) studying at a Swiss university of applied sciences at the end of their last semester. Follow-up was 1 year later, after entering the health care workplace.

### Population and sample

**Fig. 1:**
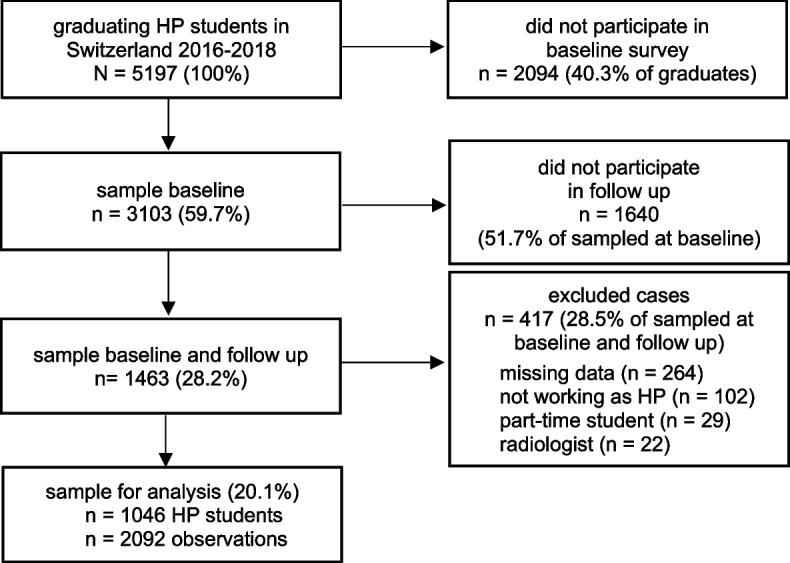
Population, return rates, and cases lost / excluded

The target population was all full-time HP students obtaining a bachelor’s degree at a Swiss university of applied sciences in 2016, 2017, and 2018. We derived data from the National Graduate Survey of Health Professionals from Universities of Applied Sciences (Nat-ABBE), a nationwide census survey of final year HP students. A total of 5197 final year HP students were asked to complete the questionnaire at the end of their sixth semester. Figure 1 shows the response rates and the cases lost and excluded from the analysis.

We excluded the following groups from this sample: students who had missing values for all variables used in the analyses, students of medical radiology because this subject can only be studied in the French-speaking part of Switzerland, part-time students because they were already working in the health care system during their studies, and HP students who were not working in the health care sector one year after graduation. We did not consider the latter as HP because their professional activity is not known, and they did not answer questions about their health status. The final sample for this study included 1046 HP and a total of 2092 observations.

### Data collection and data management

HP students were informed about the National Graduate Survey of Health Professionals (Nat-ABBE) during a class at the end of the last semester. This survey included questions about education, career expectations and plans, and questions about health. Subsequently, the HP students received an email inviting them to participate in this online survey; the participation was voluntary, and students were assured that their data would be kept confidential. In the baseline survey, participants were asked to leave an email address where they could be reached after graduation. Participants gave informed consent for the use of their data in the online questionnaire. One year after graduation, the health professionals were invited by email to participate in the follow-up survey. The survey was conducted by the Quality and Evaluation Unit of the Department of Health of the Zurich University of Applied Sciences. The data were anonymized and stored in accordance with the university’s security regulations. Data collection for the baseline started in the summer of 2016 for a first cohort of students and was repeated in 2017 and 2018 for two further cohorts. The final survey for the one-year post-graduation follow-up took place between summer 2019 and ended in May 2020, one year after the last student of the third cohort graduated.

### Measurement of self-reported musculoskeletal pain and attribution to studies or work

The Nat-ABBE online questionnaire contained a list of health problems, including low back pain, neck pain, pain in arm/hands, and pain in legs/feet. These items were taken from the Swiss Health Survey. The Swiss Health Survey is conducted by the Swiss Federal Statistical Office and is repeated every 5 years (since 1992) based on the Federal Statistics Act of 1992. Participants were asked the following question: “In the past year, did you have one or more of the following health problems?”. Answers were recorded on a four-point ordinal scale (no, rarely, occasionally, often). To make the results more comparable to other studies, we derived a subject-specific binary outcome for low back pain, neck pain, pain in arms/hands, and pain in legs/feet (yes/no), indicating the presence of any pain frequency (rarely, occasionally, often) or the absence of pain, with the category “no”.

If pain was reported in the online questionnaire, an additional question was asked for the causal attribution of this pain: “Do you think that these complaints are related to your studies/ to your work?” The answers were: no, partly, yes.

### Statistical analyses

We used Stata 15.1 (StataCorp, College Station, TX, USA) for all statistical analyses. Of the 2092 observations which were included in the analyses, complete data for all variables were available for 2024 observations (96.75%). Missing values occurred in 60 cases for a single variable (2.87%), 5 cases had 2 missing values (0.24%) and 3 cases had 4 missing values (0.14%). Missing values were most common in the age variable (*n* = 22, 1.05%). Visual pattern analysis and cross-tabulation of missing variables showed no systematic patterns in the missing data. Participant characteristics were analyzed using descriptive statistics with mean values (including standard deviation), minimum and maximum values, or, in the case of factor variables, with absolute and relative frequencies. We used McNemar’s χ^2^-Test to assess differences in the pain experience of HP students between baseline and follow-up. The McNemar’s is used in repeated measures to test the consistency of responses between two variables. Generalized estimating equation (GEE) models of the binomial family with log links were used to estimate the adjusted prevalence of pain in HP students and the corresponding differences between professional groups. Corresponding normal-based 95% confidence intervals and Z-statistic based p-values were derived using the bootstrap method with 1000 replications. We adjusted for gender and age, centered on the mean. We also used cumulative odds models, adjusting for clustering to assess pain attribution in HP students. Statistical significance was set at *p* < 0.05.

## Results

### Demographic characteristics of HP sample

The demographic characteristics of the 1046 participants are shown in Table 1.

**Table 1:** Demographic characteristics, health professionals in Switzerland; *N* = 1046

Characteristic	N	%
**Age at baseline:** Mean: 25.0; Median: 24.0		
21–25	775	74.09
26–30	203	19.41
31–35	27	2.58
36–40	10	0.96
41–45	10	0.96
46 -57	10	0.96
missing	11	1.04
**Gender**:		
Men	94	8.99
Women	945	90.34
missing	7	0.67
**Professional groups**		
occupational therapy	112	10.71
nutritional sciences	83	7.93
midwifery	107	10.23
nursing	481	45.99
physiotherapy	263	25.14

### Annual prevalence of musculoskeletal pain

**Fig. 2:**
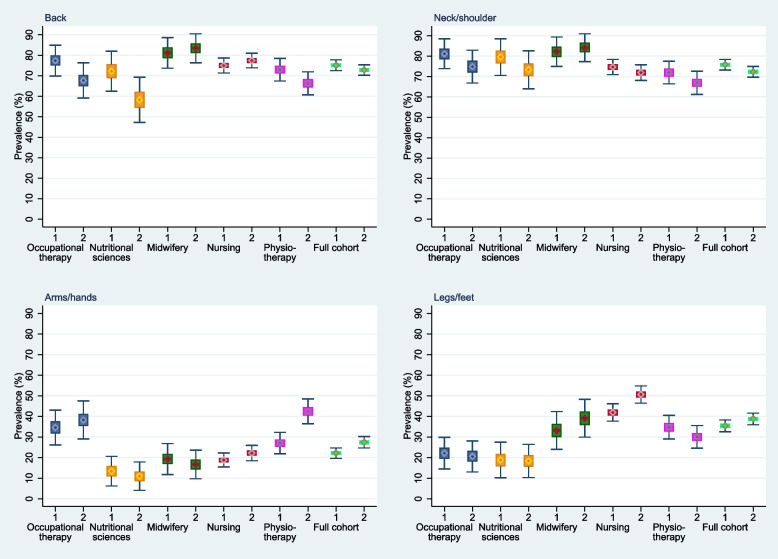
Persistence and change of musculoskeletal pain from baseline to follow-up in Swiss health professionals. Colored box comprises the interquartile range of bootstrap prevalence estimators. The white line within the box is the median bootstrap prevalence estimator. The whiskers show the bootstrap 95% normal based confidence intervals. 1 = baseline at the end of studies; 2 = follow up after one year of working in the health care workforce

Figure 2 gives an overview of the four types of musculoskeletal pain considered at baseline (1) and follow-up (2). The results were estimated by bootstrapping, adjusting for gender and age. The white line in the box is the median bootstrap prevalence estimate. The colored box shows the interquartile range of bootstrap prevalence estimates. The whiskers show the bootstrap 95% confidence intervals based on the normal distribution.

At both time points, the prevalence of low back and neck/shoulder pain is higher than the prevalence of pain in arms/hands and legs/feet. The prevalence of low back pain and neck/shoulder pain for the whole cohort decreases slightly (not statistically significant) but remains high, with proportions of 73% and 72.4% for the latter, respectively, between baseline and follow-up. The prevalence of pain in arms/hands and legs/feet in the whole cohort is higher at follow-up with proportions of 27.6% and 39.2% respectively for the latter. Midwives have the highest prevalence for low back pain and neck/shoulder pain at baseline and follow-up. Occupational therapists and physical therapists had the highest prevalence of arm/hand pain at both time points. Nurses were most likely to report leg/foot pain at baseline and follow-up. The largest increase in arm/hand pain was found among physiotherapists. In the following sections, we present the detailed results for the types of pain.

### Low back pain

Table 2 shows the adjusted annual prevalence of low back pain at baseline and follow-up for health professionals in Switzerland.

**Table 2:** Low back pain: adjusted annual prevalence; mean (95% CI); *N* = 1046

	**Occupational therapy**	**Nutritional Sciences**	**Midwifery**	**Nursing**	**Physiotherapy**	**Full cohort**
**Baseline**	78.4% (70.6–86.1)	71.6% (61.7–81.5	81.3% (74.3–88.8)	76.1% (72.3–80.0)	73.3% (67.9–78.6)	75.8% (73.2–78.5)
**Follow-up**	67.6% (58.7–76.5)	60.0% (49.5–70.5)	83.2% (76.1–90.2)	78.4% (74.8–82.0)	66.8% (61.0–72.6)	73.0% (70.7–75.9)

At baseline, midwives show the highest prevalence, nutritional scientists the lowest. However, differences in the prevalence of low back pain among full-time HP students at baseline were not statistically significant.

At follow-up, there were some differences in prevalence between different professions with higher prevalence of low back pain in midwives and nurses compared to the other HP (midwifery > physiotherapy, *p* = 0.0005; midwifery > nursing, *p* = 0.0008; midwifery > nutritional sciences, *p* = 0.0002; midwifery > occupational therapy, *p* = 0.0065; nursing > nutritional sciences, *p* = 0.0010; nursing > occupational therapy, *p* = 0.0308).

Within the HP student groups, the differences between baseline and follow-up were not statistically significant at the 5% level. However, the prevalence of low back pain decreased to near significance between baseline and follow-up for occupational therapy students [-10.1% (-22.7–1.1), p = 0.0750)].

### Neck/shoulder pain

Table 3 shows the adjusted annual prevalence of neck/shoulder pain at baseline and follow-up for health professionals in Switzerland.

**Table 3:** Neck/shoulder pain: adjusted annual prevalence; mean % (95% CI); *N* = 1046

	**Occupational therapy**	**Nutritional Sciences**	**Midwifery**	**Nursing**	**Physiotherapy**	**Full cohort**
**Baseline**	81.1% (73.7–88.5)	80.2% (71.5–89.0)	82.2% (75.0–89.5)	74.2% (70.4–78.1)	72.1% (66.7–77.5)	75.7% (73.3–78.2)
**Follow-up**	74.8% (66.3–83.2)	75.3% (65.3–85.3)	84.1% (77.1–91.1)	71.5% (67.4–75.5)	67.2% (61.5–72.9)	72.4% (69.6–75.1)

At baseline, all HP show high annual prevalence of neck/shoulder pain ranging from 82.2% (midwifery students) to 72.1 (physiotherapy students), but only the difference between midwifery students compared to physiotherapy students was statistically significant (*p* = 0.0258).

At follow-up, the prevalence of neck pain was significantly higher for midwives (84.1%) than for most other HP (midwifery > nursing, *p* = 0.0027; midwifery > physiotherapy, *p* = 0.0002).

Within the HP student groups, the differences between baseline and follow-up were not statistically significant. However, in the total sample of HP students, the prevalence of neck pain decreased slightly between baseline and follow-up [-3.4% (-7.2–0.3)], reaching borderline significance (*p* = 0.0760).

### Pain in arms/hands

Table 4 shows the adjusted annual prevalence of pain in arms/hands at baseline and follow-up for health professionals in Switzerland.

**Table 4:** Pain in arms/hands: adjusted annual prevalence; mean (95% CI); *N* = 1046

	**Occupational therapy**	**Nutritional Sciences**	**Midwifery**	**Nursing**	**Physiotherapy**	**Full cohort**
**Baseline**	34.2% (25.1–43.4)	13.9% (6.1–21.7)	19.2% (12.1–26.4)	19.5% (15.9–23.1)	27.1% (21.9–32.3)	22.5% (20.0–25.1)
**Follow-up**	37.8% (28.4–47.3)	11.1% (4.1–18.2)	16.8% (9.8–23.8)	22.2% (18.4–26.0)	42.4% (36.3–48.4)	27.6% (24.8–30.3)

With a baseline prevalence of pain in the arms/hands of 34.2% and 27.1%, respectively, occupational therapy students and physiotherapy students showed a higher prevalence compared to most other professions (occupational therapy > midwifery, *p* = 0.0115; occupational therapy > nursing, *p* = 0.0028; occupational therapy > nutritional sciences, *p* = 0.0009; physiotherapy > nursing, *p* = 0.0182; physiotherapy > nutritional sciences, *p* = 0.0052).

At follow-up, the prevalence of pain in arms/hands was significantly higher in occupational therapists and physiotherapists compared to all other HP groups (physiotherapy > nurses, *p* < 0.0001; physiotherapy > nutritional sciences, *p* < 0.0001; physiotherapy > midwifery, *p* < 0.0001; occupational therapy > nursing, *p* = 0.0038; occupational therapy > nutritional sciences, *p* < 0.0001; occupational therapy > midwifery, *p* = 0.0007).

Within the HP student groups, the adjusted prevalence of pain in arms/hands increased in physiotherapy students [15.3% (7.3–23.2)] as well as in the total sample of full-time HP students [5.0% (1.3–8.8)] (*p* < 0.0001 and *p* = 0.0080 respectively).

### Pain in legs/feet

Table 5 shows the adjusted annual prevalence of pain in legs/feet at baseline and follow-up for health professionals in Switzerland.

**Table 5:** Pain in legs/feet: adjusted annual prevalence; mean (95% CI); *N* = 1046

	**Occupational therapy**	**Nutritional Sciences**	**Midwifery**	**Nursing**	**Physiotherapy**	**Full cohort**
**Baseline**	22.5% (14.8–30.3)	19.0% (10.1–27.9)	33.0% (24.0–42.0)	42.8% (38.3–47.2)	34.8% (29.0–40.7)	35.6% (32.9–38.5)
**Follow-up**	20.9% (13.1–28.8)	18.5% (10.0–27.1)	39.3% (30.2–48.4)	52.2% (47.7–56.8)	29.9% (24.5–35.3)	39.2% (36.2–42.1)

On the one hand, nursing students showed a higher annual prevalence of pain in legs/feet at baseline then most of the other professions (nursing > physiotherapy, *p* = 0.0413; nursing > occupational therapy, *p* < 0.0001; nursing > nutritional sciences, *p* < 0.0001). On the other hand, the prevalence of pain in the legs/feet at baseline was significantly lower for nutritional sciences and occupational therapy students, with proportions of 19.0% and 22.5%, respectively, compared to most other professions (nutritional sciences < physiotherapy, *p* = 0.0033; nutritional sciences < midwifery, *p* = 0.0349; nutritional sciences < nursing, *p* < 0.0001; occupational therapy < physiotherapy, *p* = 0.0120; occupational therapy < nursing, *p* < 0.0001).

At follow-up, nurses and midwives had the highest annual prevalence of pain in legs/feet at 52.2% and 39.3%, respectively (nursing > midwifery, *p* = 0.0111; nursing > physiotherapy, *p* < 0.0001; nursing > occupational therapy, *p* < 0.0001; nursing > nutritional sciences, *p* < 0.0001; midwifery > occupational therapy, *p* = 0.0030; midwifery > nutritional sciences, *p* = 0.0009).

Within the HP student groups, the adjusted prevalence of pain in legs/feet increased significantly in nursing students [9.5% (3.1–15.8), *p* = 0.0040].

### Individual dynamics of pain experience

Depending on the type of pain, full-time HP students in Switzerland experienced different patterns of change in pain over time (see Table 6).

**Table 6:** Individual dynamics of pain experience between baseline and follow-up; *N* = 1046

Pain symptoms	low back	neck/shoulder	arms/hands	legs/feet
yes at baseline—yes at follow-up	62.1%	61.4%	9.8%	20.6%
no at baseline—no at follow-up	13.4%	13.3%	59.5%	45.6%
yes at baseline—no at follow-up	13.4%	14.5%	12.8%	15.1%
no at baseline—yes at follow-up	11.2%	10.8%	17.9%	18.6%
McNemar’s χ^2^(1); p	2.07; 0.1498	5.78; 0.0162	8.56; 0.0034	3.72; 0.0536

The patterns for low back pain and neck/shoulder pain are similar: most students who reported low back or neck/shoulder pain at baseline still reported them at follow-up. Slightly more students experienced an improvement in their low back or neck/shoulder pain; this overall change over time was significant only for neck/shoulder pain (*p* = 0.0162).

Most full-time HP students had no pain in arms/hands, but more students experienced a change for the worse over time compared to students who had no pain in arms/hands at follow-up (*p* = 0.0034).

No pain in legs/feet at both times was the most common pattern, with the overall burden of pain in legs/feet increasing over time (borderline significance: *p* = 0.0536).

### Attribution of pain

**Fig. 3:**
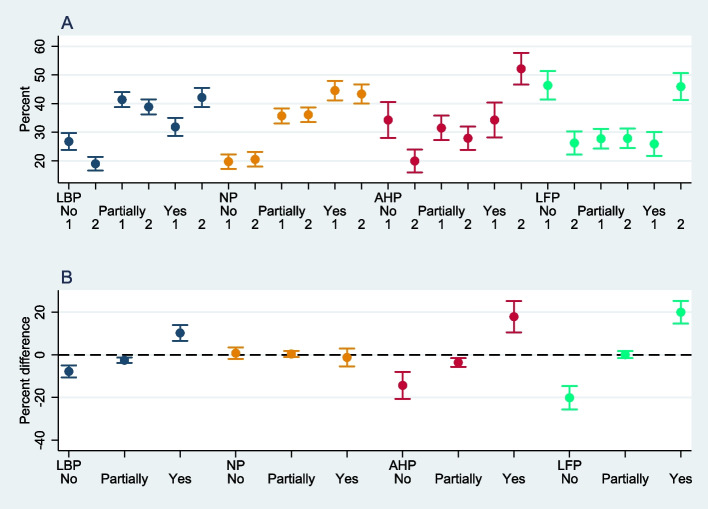
Causal attribution of musculoskeletal pain. LBP: lower back pain; NP: neck/shoulder pain; AHP: pain in arms/hands; LFP: pain in legs/feet

Figure 3 shows whether HP associate their pain completely, partially or not with study/work. The upper part of the Figure (A) shows the estimated percentage by response category (yes, partially, no) at baseline (1) and follow-up (2) with 95% confidence intervals; the lower part (B) shows the estimated percentage difference between baseline (1) and follow-up (2) by response category with 95% confidence intervals.

Low back pain in the HP study cohort in Switzerland is mainly attributed to work or studies. Only a minority of full-time HP students reported that low back pain was not related to either their studies at baseline [26.8% (23.8–29.7)] or to work at follow-up [19.0% (16.6–21.4)]. In addition, there was a significant and substantial increase at follow-up of 10.3% (6.6–14.0) in those who attributed low back pain to study/work, while the percentage of HP students who did not attribute low back pain to work or who attributed low back pain partly to work decreased by 7.8% (4.9–10.6) and 2.5% (1.4–3.7) respectively.

As with low back pain, neck pain is mainly attributed to work or studies by full-time HP students in Switzerland. Only a minority of HP students did not attribute neck pain to studies at baseline [19.7% (17.2–22.3)] or to work at follow-up [20.5% (17.9–23.1)]. With 35.7% (33.1–38.3) and 36.1% (33.5–38.7), respectively, more than a third attributed neck pain partly to work at baseline or follow-up, and a majority attributed neck pain to study/work at both times [44.6% (41.1–48.0) and 43.4% (40.0–46.7), respectively]. In contrast to low back pain, the attribution of neck pain did not change significantly over time.

As shown in Fig. 2 above, pain in the arms/hands is less common than low back pain and neck pain, but this form of pain is also mainly attributed to work or study by the HP of our cohort, with a significant increase between baseline and follow-up. At baseline, around a third of full-time HP students reported pain in the arms/hands as not related to their studies, partly related to their studies or related to studies [34.3% (28.0–40.5), 31.5% (27.2–35.8), and 34.3% (28.2–40.4), respectively]. At follow-up, the percentage of HP students attributing pain in the arms/hands to work increased by 17.9% (10.6–25.3), while those attributing pain in the arms/hands to work partly or not at all decreased by 3.6% (1.6–5.6) and 14.3% (8.0–20.6) respectively.

A similar pattern of a significantly increased proportion of HP attributing their pain to work at follow-up appears for pain in the legs/feet. At baseline, most full-time HP students did not attribute pain in the legs/feet to their studies [46.4% (41.4–51.3)], 27.7% (24.3–31.2) partly attributed pain in legs/feet to their studies, and only a minority of 25.9% (21.8–30.0) reported pain in the legs/feet to be related to their studies. At follow-up, however, a substantial majority attributed pain in the legs/feet to work [45.9% (41.2–50.6)], 27.8% (24.4–31.2) partly attributed pain in legs/feet to work, and only 26.2% (22.2–30.3) did not attribute pain in the legs/feet to work. In summary, the percentage of students who did not attribute pain in the legs/feet to work decreased significantly, while the percentage of students who attributed pain in the legs/feet to work increased.

Overall, we found that, except for neck pain, the percentage of HP students attributing pain to work had significantly increased by follow-up (Fig. 3).

### Summary of most important results

Table 7 presents the most important results of this study.

**Table 7:** Summary of major results for musculoskeletal pain in Swiss health professionals; *N* = 1046

	**low back**	**neck/shoulder**	**arms/hands**	**legs/feet**
Overall prevalence baseline/ follow-up	75.8% / 73.0%	75.7% / 72.4%	22.5% / 27.6%	35.6% / 39.2%
Substantial differences in prevalence between professional groups	highest for midwives (83.2%) and nurses (78.4%) at follow-up	highest for midwives (84.1%) at follow-up	highest for physiotherapists (42.4%) and occupational therapists (37.8%) at follow-up	highest for nurses and midwives at follow-up
Individual change of pain experience over time	little change, most common is pain at both time points (62.1%)	little change, most common is pain at both time points (61.4%)	substantial increase in physiotherapists (27.1%—> 42.4%)	significant increase in nurses (42.8%—> 52.2%)
Partial or total causal attribution of pain to studies at baseline/ to work at follow up	73.2% / 81.2%	80.3% / 79.5%	65.8% / 80.1%	53.6% / 73.7%

Pain in the lower back and neck/shoulder was common among HP, with midwives being most susceptible to this pain. Individual experiences of pain in the lower back and neck/shoulder were often constant over time, and more HP experienced a change for the worse than an improvement. Most HP attributed some or all the causes of lower back pain and neck/shoulder pain to their studies or work (Table 7). Pain in arms/hands and legs/feet was less common. Physiotherapists and occupational therapists were more likely to report pain in arms/hands than other professional groups; nurses and midwives were more likely to report in legs/feet than other HP with significant increase after one year of working in the health care sector. The attribution of the causes of pain in arms/hands and legs/feet was ambiguous at baseline; after working one year in the health care sector, HP attributed their pain in arms/hands and legs/feet more often to their work.

## Discussion

In this follow-up study, we investigated the prevalence and individual course of musculoskeletal pain in HP at the transition from study to work in Switzerland. Full-time HP students reported their pain using an online questionnaire at the end of their studies and one year after entering the healthcare workforce. We were particularly interested in the question of whether musculoskeletal pain in HP is already present during studies or only occurs in professional life.

The results strongly suggest that low back pain and neck/shoulder pain in HP already occur during their studies: 75% of the participants reported low back pain and or neck/shoulder pain at baseline; low back pain and neck/shoulder pain were present in 62% and 61%, respectively, at both time points (see Table 7: Overall prevalence and individual change over time). This was true for all professional groups in our study.

In contrast, the prevalence of pain in arms/hands and legs/feet is generally lower: 22.5% and 35.6% respectively. The prevalence of pain in arms/hands increased significantly at follow-up, especially among physiotherapists. The prevalence of pain in legs/feet increased significantly among nurses after they started working. This suggests that work-related factors are responsible for this pain in physiotherapists and nurses. This is supported by the attributions that physiotherapists and nurses make for their pain: They associate their pain more strongly with their professional life at follow-up than with their studies at the baseline.

### Differences between professional groups

Midwives and nurses have the highest prevalence of low back and neck pain. These two professions perform unfamiliar and physically demanding tasks such as bending and lifting during internships and after graduation, which may explain the difference compared to the other HP groups.

Occupational therapists and physiotherapists had the highest prevalence of pain in the hands/arms and a significant increase between baseline and follow-up. This may be explained by the higher demands on the arms and hands, for example from manual therapies, which are common in these professions.

Nurses and midwives were most affected by pain in the legs/feet with a significant increase between baseline and follow-up. This is probably related to prolonged standing and walking, which is common in the daily routine of these professions. In Switzerland, about 78% of nurses spend at least half of their working time standing, and 65% must do so for at least three-quarters of the time. These rates are higher than in other professions: only 46% of medical doctors and 59% of other health professionals spend at least half of their work time in a standing position [12]. Although many nurses experience leg/foot pain, it receives little attention compared to other musculoskeletal disorders. In a recent systematic review of interventions to prevent musculoskeletal injuries in nurses [3], none of the 20 included studies focused on the lower limbs. Most of the interventions were aimed at preventing back pain. Almost ironically in this context, an intervention study investigating the effects of unstable footwear focused on low back pain and disability as outcome variables [3].

### Causal attribution of pain

It is not possible to deduce the causes of low back/neck pain from the available data. However, the majority of HP attribute their low back pain and neck/shoulder pain completely or at least in part to their studies/work. In the last year of their studies, students write their bachelor’s thesis, which involves long hours of computer/laptop work, and they are often in an internship.

Full-time HP students did not associate their pain in arms/hands and legs/feet as clearly with their studies as they did with low back pain and neck/shoulder pain. This changed after one year of work: 80.0% associated pain in arms/hands and 73.7% associated pain in legs/feet fully or at least partly with their work.

The stronger association of these pains with work in the health sector corresponds to the increase in these complaints after starting work in the health sector. The work of physiotherapists and occupational therapists requires the use of hands and arms. The lower back, shoulders/neck and legs/feet of midwives and nurses are exposed to high levels of strain in their professional lives. It is understandable that HP, after their office-based studies, associate the causes of these complaints with the strenuous work in hospitals or outpatient clinics.

### Comparison of prevalences with previous studies

Previous studies have reported the following one-year prevalences of musculoskeletal pain in HP:

- low back pain: 55.0% to 73.1% [19–22]
- neck/shoulder pain: 13.0% to 96.0% [3, 19–22]
- pain in arms/hands: 14.0% to 33.6% [19, 20, 22]
- pain in leg/feet: 36.0%–65.7% [20, 22]

A direct comparison of our results with other studies is not possible because of different measurement methods and more heterogeneous age groups in other studies. Also, the range of prevalence in the studies found is very wide, especially for neck/shoulder pain. Nevertheless, our results are within the range of previous studies, which we take as an indication of the trustworthiness of our data.

## Recommendations

For most health professionals, low back pain and neck/shoulder pain start during their studies and continue into the first year of work, and these symptoms are mainly attributed to study or work. The prevalence of low back pain and neck/shoulder pain is alarmingly high, considering that this study mainly examined young health professionals at the beginning of their careers. Moreover, if we consider the results of clinical follow-up studies [23–28], which mostly suggest a chronic or intermittent course for low back pain and neck/shoulder pain, this gives a poor prognosis for the future if so many health professionals start their career with low back pain and neck/shoulder pain. Low back pain and neck/shoulder pain are therefore not only a burden on the individual, but also a public health problem in two ways: First, the high prevalence represents a risk and burden for the general public in terms of health insurance costs, occupational insurance costs, and increasing tax transfers. Second, in view of the shortage of health professionals, it is a threat to the health care system: the premature retirement of qualified health professionals due to health problems compromises the provision of health care for the whole population, i.e. a potential shortage of essential health services.

In addition to this, full-time HP students and HP strongly associate their low back pain and neck/shoulder pain with study and work, respectively. This negative connotation can prevent positive feelings about the job and reduce motivation to continue working for as long as possible. It is therefore important to avoid this negative connotation. Computer work and physically demanding tasks cannot be avoided in the health professions. Therefore, health self-care for these known risks must become an integral part of the training of health professional. The period of study has great potential for the prevention of these health problems. Universities have the know-how and the infrastructure to integrate the prevention of low back pain and neck pain into their curricula and to implement preventive measures during the studies.

Pain in arms/hands and pain in legs/feet are more common at work than at university and are mainly attributed to work. There are differences between different professions and more research is needed to better understand the causes and to develop interventions to reduce pain in legs/feet for nurses and midwives and pain in arms/hands for physiotherapists and occupational therapists.

### Strengths, weaknesses, and limitations of this study

The longitudinal design is a major strength of this study, with its two repeated intra-individual measures of pain. This allowed us to show that many HP suffer from low back pain and neck pain during their studies and that these complaints are not acquired during their working lives. However, there are several limitations to the study. Firstly, our study only looked at the presence of pain. Other relevant factors, such as pain intensity and chronicity, could not be considered because they were not part of the questionnaire. Second, the dropout rate was relatively high, i.e. more than 50% of the students who completed the baseline questionnaire did not attend the follow-up and were not included in the study. Consequently, selective response bias due to missing data may have influenced our results. Although our sensitivity analyses, in which we assessed pain at baseline between participants and non-participants at follow-up (adjusted for age, gender, and type of health profession), did not show significant differences, selective response bias may still be present. Third, all data are self-reported. As such, they may be subject to recall bias, social desirability bias, or may depend on social and professional experiences and meanings derived from the respondents’ social environment (“Lebenswelt”).

## Conclusions

Musculoskeletal pain is a major issue among students and young HP. Low Back and shoulder–neck pain already occurs during studies, while pain in arms/hands and legs/feet tend to occur after entering professional life. The results call for research into the causes of these complaints so that empirically based preventive measures can be taken. Further research on the incidence and course of musculoskeletal pain in students of other fields of study, and working adolescents is warranted to gauge the scope of this problem.

## Data Availability

The dataset analyzed during the current study is available online: https://zenodo.org/record/7123425.

## Abbreviations

HP: Health professionals
HP students: Students of health professions
Nat-ABBE: National Graduate Survey of Health Professionals
